# Analysis of Retinal Microstructure in Eyes with Dissociated Optic Nerve Fiber Layer (DONFL) Appearance following Idiopathic Macular Hole Surgery: An Optical Coherence Tomography Study

**DOI:** 10.3390/jpm13020255

**Published:** 2023-01-30

**Authors:** Shucheng He, Xin Ye, Wangli Qiu, Shangchao Yang, Xiaxing Zhong, Yiqi Chen, Rui He, Lijun Shen

**Affiliations:** 1 National Clinical Research Center for Ocular Diseases, Eye Hospital, Wenzhou Medical University, Wenzhou 325027, China; 2 Department of Ophthalmology, Zhejiang Provincial People’s Hospital, Hangzhou 310014, China; 3 Computer Science & Software School, Hangzhou Dianzi University, Hangzhou 310018, China

**Keywords:** dissociated optic nerve fiber layer, retinal dimples, internal limiting membrane, idiopathic full-thickness macular hole, optical coherence tomography

## Abstract

(1) Purpose: This study aimed to evaluate morphological changes of the retina in eyes with dissociated optic nerve fiber layer (DONFL) appearance following internal limiting membrane (ILM) peeling for full-thickness idiopathic macular hole (IMH) on spectral-domain optical coherence tomography (SD-OCT). (2) Methods: We retrospectively analyzed 39 eyes of 39 patients with type 1 macular hole closure after a vitrectomy with ILM peeling procedure at a six-month minimum postoperative follow-up. The retinal thickness maps and cross-sectional OCT images were obtained from a clinical OCT device. The cross-sectional area of the retinal nerve fiber layer (RNFL) on cross-sectional OCT images was manually measured by ImageJ software. (3) Results: The inner retinal layers (IRLs) thickness thinned down much more in the temporal quadrant than in nasal quadrants at 2 and 6 months postoperatively (*p* < 0.001). However, the cross-sectional area of the RNFL did not change significantly at 2 and 6 months postoperatively (*p* > 0.05) when compared to preoperative data. In addition, the thinning of the IRL did not correlate with the best-corrected visual acuity (BCVA) at 6 months postoperatively. (4) Conclusions: The thickness of the IRL decreased in eyes with a DONFL appearance after ILM peeling for IMH. The thickness of the IRL decreased more in the temporal retina than in the nasal retina, but the change did not affect BCVA during the 6 months after surgery.

## 1. Introduction

**Figure 1:**
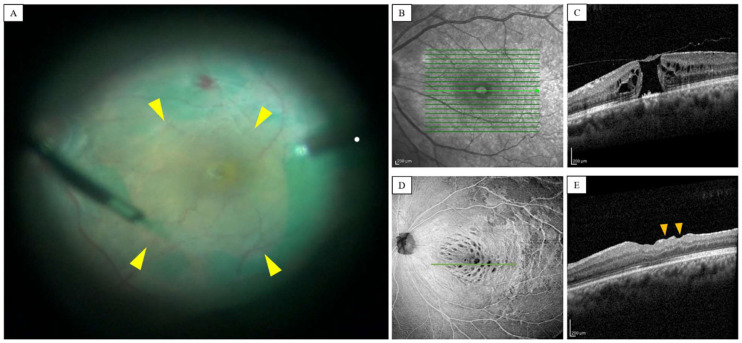
Left eye of a 61-year-old man treated with vitrectomy and ILM peeling for IMH. (**A**). The ILM around the macular hole was removed using pinch-and-peel technique after indocyanine green staining. The yellow arrows represent the extent of the inner limiting membrane peeling. A full-thickness macular hole was observed in fundus image (**B**) and OCT B-scan (**C**) before the surgery. The green line with the arrow in (**B**) indicates the scan line for the B-scan OCT images in (**C**). En face OCT images (**D**) at four-month postoperatively showed a clear CMDS appearance (i.e., concentric dark spots) inside the ILM peeling area. And some focal dimples corresponding to dark spots on green line in (**D**), were visible in the RNFL on B-scan OCT images in (**E**) (yellow arrows).

Idiopathic macular hole (IMH) is a retinal disease that seriously threatens patients’ vision and quality of life [1,2]. The mechanism of IMH involves the disruption or loss of the Müller cell cone in the fovea, and this is caused by the vitreoretinal traction perifovea [3,4,5] (see Figure 1C). A vitrectomy with the internal limiting membrane (ILM) peeling procedure has been proven to promote postoperative macular hole closure and improve visual function in previous studies [6,7]. However, ILM plays an important role in homeostasis and maintenance of inner retinal layers, as it is the basal membrane of Müller cells. Moreover, in some cases, the removal of the ILM results in various complications, such as swelling of the arcuate retinal nerve fiber layer (SANFL), macular retinal displacement, dissociated optical nerve fiber layer (DONFL), etc. [8]. The study on the morphological changes of the retina after the removal of the ILM in eyes with macular hole can help us to further elucidate the potential damage caused by the surgery.

Optical coherence tomography (OCT) is a non-invasive optical imaging technique that enables in vivo imaging of the structural morphology and blood flow (OCT angiography) of the retina in both human and a variety of veterinary species [9,10]. DONFL appears as dimples (see Figure 1E) in the inner retinal layers on cross-sectional OCT images and as concentric macular dark spots (CMDS) in en face OCT images [8,11] (see Figure 1D). Several studies have shown that a DONFL appearance occurs without the loss of the nerve fiber layer and argued that DONFL is caused by the rearrangement rather than the loss of optic nerve fibers after ILM peeling [12,13]. However, some scholars believe that DONFL involves damages in the deeper area under the retinal nerve fiber layer (RNFL), such as the ganglion cell layer–inner plexiform layer (GCL–IPL) complex [14,15]. To date, the pathogenesis of DONFL and the role of the retinal layer have not been well clarified.

Recently, with the help of high-resolution OCT, the structure of the retina could be analyzed layer by layer, using thickness maps. Several studies have shown that ILM peeling in IMH causes progressive thinning of some retina layers [16,17,18]. However, it is not known whether this retinal-layer thinning is associated with the formation of DONFL, and there is a lack of longitudinal observations of the retinal structure in DONFL patients after ILM peeling. The study of the retinal layer changes in DONFL may help to shed light on the formation mechanism of DONFL and its potential damage to vision.

Given the high incidence of DONFL after ILM peeling in IMH [19], this study aimed to evaluate the retinal structure in eyes with DONFL after ILM peeling for IMH through long-term follow-up to explore the mechanism of DONFL formation. We also analyzed the correlation between the change of retinal layers and visual acuity after surgery. We hope this study can help to provide more information for clinical practice to improve the surgical procedures.

## 2. Methods

### 2.1. Ethical Approval

This was a retrospective study that adhered to the tenets of the Declaration of Helsinki. Institutional Review Board (IRB) approval was obtained from the Affiliated Eye Hospital of Wenzhou Medical University, and informed consent was obtained from all subjects.

### 2.2. Inclusion and Exclusion Criteria

Inclusion criteria were eyes diagnosed as IMH and underwent ILM peeling between January 2017 and September 2020. All the included eyes achieved a type 1 closure pattern and at least 6 months of postoperative follow-up. The type 1 closure of IMH was defined as some reconstitution of the banded anatomy [20]. All the included eyes’ pre- and postoperative fundus photographs, as well as OCT scans with a minimum follow-up of six months, were accessed. The size of the macular hole was measured as the minimum hole width or the narrowest aperture size in the middle retina on cross-sectional OCT images, as defined by The International Vitreomacular Traction Study (IVTS) Group on initial presentation [21]. Eyes with other severe vitreoretinal diseases (e.g., glaucoma, retinitis pigmentosa, diabetic retinopathy, and pathological myopia), a history of the previous vitrectomy, and poor OCT image quality were excluded from the study.

### 2.3. Surgical Procedure

The standard three-port pars plana vitrectomies were performed in all patients by a surgeon, using a 23-gauge transconjunctival vitrectomy system (see Figure 1A). Phacoemulsification with intraocular lens implantation would be performed if the cataract was severe enough to interfere with intraocular surgery. Triamcinolone acetonide was used to assist posterior vitreous detachment (PVD). ILM was removed around the macular hole in 2–3 disc diameters, using a pinch-and-peel technique with forceps stained with 0.02 mL of indocyanine green (0.025 mg/mL) (see Figure 1A). Sterile air was filled in the vitreous cavity following the exchange of gas and liquid. For a minimum of seven days after surgery, all patients had to remain facedown.

### 2.4. Data Collection

Information collected included demographic data (e.g., age and sex), laterality, pre-and postoperative best-corrected visual acuity (BCVA), lens status, B-scan SD-OCT images (Spectralis HRA, Heidelberg Engineering, Germany), and en face OCT images (RTVue, Optovue, San Francisco, CA, USA). The B-scan mode included a linear horizontal scan of 20° × 15° (6.6*4.9 mm). BCVA measurements were performed by using the Snellen chart and were converted to units of logarithms of the minimum angle of resolution (logMAR) for statistical analyses. The postoperative parameters were analyzed two and six months after surgery.

**Figure 2:**
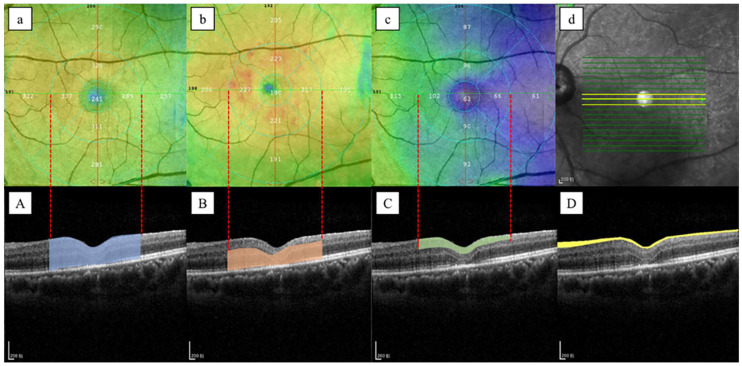
Left eye of a 57-year-old woman treated with vitrectomy and ILM peeling for IMH. (**a**–**c**). The thickness of TRL, ORL, and IRL in different parafoveal regions was automatically identified using the Early Treatment Diabetic Retinopathy Study (ETDRS) grid and measured by RTVue XR OCT. (**d**). The B-scan OCT images of the fovea, the closest superior to the fovea, and the closest inferior to the fovea were used for the measurement of RNFL cross-sectional area. (**A**). The TRL was defined as the area between the ILM and the RPE as the area shown in blue. (**B**). The ORL was defined as the area from the inner nuclear layer to the RPE as the area shown in orange. (**C**). The IRL was defined as the area from the ILM to the inner plexiform layer as the area shown in green. (**D**). RNFL cross-sectional area (i.e., yellow section) in B-scan OCT images was measured manually using ImageJ software.

On en face OCT images, all postoperative images were assessed to determine whether or not inner retinal dimples were present. The retinal layers were automated segmented with the 6*6 mm scan mode in the macular region, using the RTVue XR OCT. One retinal specialist would make manual adjustments if the segmentation was improper before further retinal thickness measurement. Then the mean thickness of the total retinal layer (TRL), the outer retinal layer (ORL), and the inner retinal layer (IRL) of the parafoveal area (i.e., the area with an inner diameter of 1mm and an outer diameter of 3 mm) were automated measured with embed software and exported for further analysis (see Figure 2). TRL was defined as the retina layer between the ILM and the retinal pigment epithelium (RPE), the ORL was defined as the retina layer from the inner nuclear layer to the RPE, and the IRL was defined as the retina layer from the ILM to the inner plexiform layer. All the thickness data in each quadrant were exported for further analysis. In addition, the ImageJ (software version 1.52; National Institutes of Health, Bethesda, MD, USA) was used to quantitatively assess the cross-sectional area of the RNFL. This measurement method has been proven effective in previous studies [13]. In brief, three horizontal B-scan OCT images (i.e., subfoveal, the closest superior, and inferior to the fovea) with 6 mm scan length and 0.25 mm intervals at each follow-up visit were selected. The area of RNFL in each image was measured manually, using the ImageJ program by one observer, and the area was quantified in pixels (see Figure 2). Fifty OCT images were chosen randomly from the collected images to determine the reproducibility of the measurement, and two observers were asked to evaluate them individually. To determine the repeatability of the measurement, Observer 1 was asked to measure the same 50 images one week after the first measurement and was blind to the previous scores.

### 2.5. Statistical Analysis

Statistical analyses were conducted with statistics software (IBM SPSS, version 26; IBM Corp., Armonk, NY, USA). The normality of data distribution was confirmed by using the Shapiro–Wilk test. The normally distributed variables were expressed as mean ± standard deviation, and the non-normally distributed variables were expressed as median (inter-quartile range). Repeated measures ANOVA was used to evaluate the changes in different retinal layers over time. Considering inter-group correlations, generalized estimating equations (GEEs) were used to analyze differences in the reduction of retinal layer thickness in different quadrants. Multiple comparisons between the groups within each analysis were performed by using the Bonferroni correction test. Correlation tests were conducted by using Spearman’s correlation test. A *p*-value of less than 0.05 was considered statistically significant.

## 3. Results

### 3.1. Subject Characteristics and Incidence of DONFL

The study included 39 eyes from 39 patients with at least a 6-month follow-up (9 men and 30 women, ages 25 to 84, mean age, 59.487 ± 10.406 years). The axial length was, on average, 24.318 ± 1.914 mm. Except for the eye of a 24-year-old woman who had no diagnosis of cataract, all of the included eyes underwent phacoemulsification and intraocular lens implantation. In total, there were 8 (20.51%) eyes with a small macular hole, 8 (20.51%) eyes with a median macular hole, and 23 (58.97%) eyes with a large macular hole. Mean preoperative BCVA was 0.929 ± 0.494 logMAR units, and BCVA 6 months postoperatively was 0.311 ± 0.283 logMAR units. The improvement in BCVA from the baseline was statistically significant (*p* < 0.001). At the initial checkup two months following surgery, 30 eyes (76.92%) had DONFL in all four quadrants, compared to 9 eyes (23.08%) that had DONFL exclusively in the temporal quadrant. DONFL was noticed throughout the course after 6 months following surgery in each of the four quadrants (100%). Table 1 gives all the information.

### 3.2. Retinal Thickness Decreased in DONFL after Surgery

**Figure 3:**
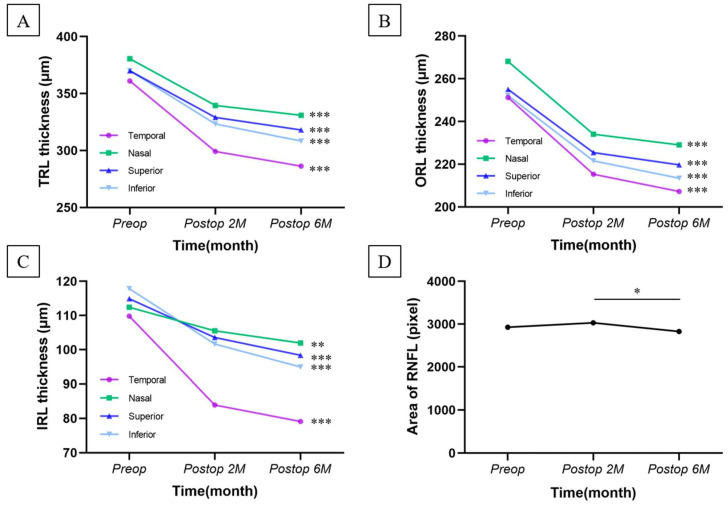
Trends of changes and regional differences in the different retinal layers of the patients with DONFL during postoperative follow-up. (**A**–**C**). The thickness of TRL, ORL, and IRL decreased significantly in all quadrants. (**D**). The area of RNFL increased slightly at 2-month operatively with no significant difference, then decreased significantly between 2 months operatively and 6 months operatively, but there is still no significant difference compared to the preoperative. * Statistically significant difference(*p* < 0.05). ** Statistically significant difference(*p* < 0.01). *** Statistically significant difference(*p* < 0.001).

**Figure 4:**
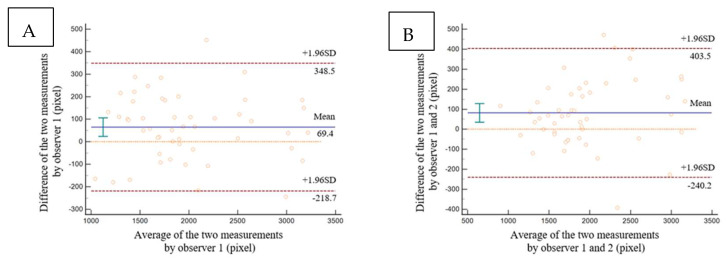
Results of intra-observer and inter-observer agreement for RNFL measurement. (**A**) Results of intra-observer agreement for RNFL measurement. The abscissa was the average of the two measurements from observer 1, and the ordinate was the difference of the two measurements from the same observer. (**B**) Results of inter-observer agreement for RNFL measurement. The abscissa was the average of the two measurements from observer 1 and observer 2, and the ordinate was the difference between the two measurements from observer 1 and observer 2.

The retinal thickness analysis was performed in all eyes. The mean thickness of the layers for each quadrant during the follow-up is listed in Supplementary Table S2. The trends of retinal thickness in each quadrant are shown in Figure 3. The thickness of TRL and ORL in all quadrants was decreased at the 6-month postoperative follow-up (*p* < 0.001). Moreover, the IRL thickness in all quadrants decreased at 6-month postoperative follow-up (*p* < 0.001 for temporal, superior, and inferior; *p* = 0.008 for nasal). The cross-sectional area of the RNFL increased slightly from 2945.436 ± 559.580 pixels preoperatively to 3048.222 ± 572.067 pixels at 2 months postoperatively, but the change was not statistically significant (*p* = 0.623). Four months later, the cross-sectional area of the RNFL later decreased to 2841.256 ± 590.553 pixels at 6 months postoperatively (*p* = 0.044), but the finding was not statistically different from the baseline (*p* = 0.463) (as shown in Figure 3). The intraclass correlation coefficient (ICC) of the measurements for intra-observer repeatability and inter-observer reproducibility were, respectively, 0.971 and 0.960. The results of the Bland–Altman analysis showed that the measurements had good intra-observer repeatability and inter-observer reproducibility (as shown in Figure 4). The intervals between the 95% limits of agreement were relatively small and suitable for clinical evaluation (inter-observer, −218.7 to 348.5; intra-observer, −240.0 to 403.5). This suggests that the RNFL area measurement is generally accurate and trustworthy.

### 3.3. The Extent of Thinning in the Layers of the Retina

We further compared the extent of thinning in ORL and IRL in each quadrant during the 6-month follow-ups (Table 2). The result showed that there was no significant difference in the reduction of ORL thickness between the temporal and nasal quadrants at the 2- and 6-month follow-ups (*p* = 1.000). There was also no significant difference in the reduction of ORL thickness between the superior and inferior quadrants at the 2- and 6-month follow-ups (*p* = 1.000). At the 2- and 6-month follow-ups, it was discovered that the IRL thickness decreased more in the temporal quadrant than in the nasal quadrants, and the differences were statistically significant (*p* < 0.001). However, no statistical difference was found between superior and inferior quadrants in IRL thickness decrease (*p* = 0.171 for 2 months, *p* = 0.077 for 6 months). The retinal thickness at baseline was analyzed, and the result (Supplementary Table S3) showed that the nasal ORL was significantly thicker than the temporal ORL (*p* < 0.001). However, there was also no significant difference between the superior and inferior ORL thickness (*p* = 1.000). As for IRL, the temporal IRL was thinner than the nasal IRL, but there was no significant difference (*p* = 1.000). The thickness of superior IRL and inferior IRL also showed no difference (*p* = 1.000).

### 3.4. Correlation between Retinal Layers Thinning and BCVA

In order to further explore whether the thinning in the IRL and ORL was correlated with the postoperative BCVA in eyes with DONFL, we analyzed the correlation between the extent of thinning in both layers and BCVA 6 months after surgery, using the Spearman correlation test. The results indicated that BCVA did not correlate with IRL or ORL thinning in each quadrant (Table 3) and also did not correlate with the mean thinning of IRL or ORL.

## 4. Discussion

Previous studies have shown a strong link between ILM peeling and the formation of DONFL [14,22,23]. Mitamura et al. [12] found that DONFL was present in 62.2% of the ILM peeling group limited in areas with ILM peeling and 0.0% in the non-ILM peeling group. Another study reported that the incidence of DONFL was as high as 100% after idiopathic full-thickness macular hole (FTMH) surgery [11]. Most studies consider defects in the IRL in DONFL after ILM peeling to be limited to RNFL changes and consider DONFL to be a reorganization of RNFL. However, some research argued that DONFL involves damages in IRL rather than RNFL rearrangement only [14].

In this study, we analyzed 39 of 39 DONFL eyes and found significant reductions in ORL and IRL thickness in all quadrants at 6 months postoperatively. A further analysis showed that the thickness of the temporal IRL decreased more than the IRL thickness in the nasal quadrant; these findings are similar to the findings of other researchers. For example, Tada et al. [18], by using retinal thickness maps, found that ILM peeling resulted in progressive thinning of the temporal inner retina at 6 months postoperatively, but no significant changes were found in the nasal inner retina. In addition, the distribution pattern of changes in IRL thickness is highly similar to what we have in our previous quantitative study of DONFL: the severity of DONFL was greater on the temporal side than in the other quadrants [24]. Given that DONFL is an inner retinal appearance and that the distribution of changes in ORL did not have a similar pattern to that of DONFL, we speculate that the formation of DONFL might be related to a reduction in IRL thickness. As an indirect indicator of retinal depression, the cross-sectional area of RNFL, compared to preoperative data, increased within 2 months postoperatively and decreased at 6 months postoperatively, yet these changes did not reach statistical significance. These changes in the RNFL were also observed by Clark et al. [25]; the study found swelling of the inner retina within 1 week to 1 month after ILM peeling. Using intraoperative OCT, Runkel et al. [19] found an increase in intraoperative nerve fiber layer thickness and a significant association with the development of postoperative DONFL appearance. Generally, the nerve fiber layer swelling disappeared after an average of 2 months postoperatively, so the reduction in RNFL cross-sectional area observed in this study between 2 and 6 months after surgery may be related to the disappearance of swelling, and the RNFL may be rearranged rather than becoming defective in the development of DONFL.

Given that the RNFL area did not alter considerably in our investigation, a drop in IRL thickness would have happened in deeper layers, such as GCL–IPL, after ILM peeling. Demirel et al. [15] also discovered that, following ILM peeling, the GCL–IPL thickness decreased, and the GCL thinning was particularly noticeable in the inner retinal dimples. According to researchers, retinal ganglion cell (RGC) mortality may not be the source of GCL thinning in the foveal region since, if RGC death resulted in the emergence of DONFL, there should be a corresponding arcuate loss of nerve fibers and a visual-field deficiency. However, previous studies have not observed any visual-field defects in DONFL patients [22]. Therefore, the thinning of GCL in the depressed area may be related to the loss or damage of Müller cells in this area [26]. The endfeet of Müller cells participated in the formation of the ILM [27]. Stripping the ILM may deprive the Müller cells of their endfeet function, causing the remainder to degenerate.

Another hypothesis in the pathogenesis of DONFL is related to anoikis in RGCs [28]. Anchorage-dependent cells that undergo dissociation from the surrounding extracellular matrix experience a type of apoptosis known as anoikis [29]. As we all know, ILMs are formed by the foot processes of Müller cells, and the expression of integrins has been detected in the ILM [30]. Moreover, since the focal adhesion kinase is expressed in RGCs [31,32], RGCs might act as an anchorage-dependent cell adjacent to the ILM, and ILM peeling might trigger the anoikis in RGCs. Recent studies have suggested that there may be undifferentiated cells in the fovea, such as retinal stem cells [33]. This sets the foundation for apoptosis, as apoptosis occurs in a large number of cells during the integration of early generated neurons [34,35]. It has been shown that ganglion cells express E-cadherin during embryogenesis [36], and E-cadherin is known to be a glycoprotein associated with anoikis. βA3/A1 crystallin, a protein associated with apoptosis, has also been discovered [37]. RGCs instead of Müller cells expresses the protein. Therefore, we speculate that the appearance of DONFL is due to ILM-peeling-induced anoikis in newly formed RGCs from retinal stem cells rather than in Müller cells. Apoptosis of these newly formed RGCs promotes a DONFL appearance and leads to a reduction in IRL thickness. In the present study, there was no correlation between IRL changes in each quadrant and postoperative BCVA 6 months after surgery. This finding can be explained by the above theory because the newly generated RGCs may not yet be integrated into the neural network and are not involved in visual function. Moreover, the cross-sectional area of RNFL did not change compared to preoperative data, and this serves as another piece of evidence for the above theory. The reason for the greater reduction in retinal thickness in the temporal IRL may be that the nerve-fiber layer is thinner on the temporal side than other sides, and the ganglion cells are less protected by RNFL, thereby leaving the area more susceptible to damages. Based on these explanations, we propose that developmental changes in DONFL may be attributed to multiple factors, such as RNFL layer rearrangements and GCL–IPL layer defects, and are closely associated with alterations in Müller cells and RGCs.

The main limitations of this study are listed as follows: (1) This study was retrospective, and the sample size of patients was relatively small. (2) The detection of visual function is relatively simple and lacks more evaluation of visual function except for BCVA. (3) This was a single-center study, and a multicentric study is necessary for further analysis.

In conclusion, there was thinning in IRL and ORL in patients with DONFL, and the IRL thinning mainly involved the temporal retina. The results show that there is no significant correlation between retinal thinning and postoperative BCVA in DONFL patients. 

## Figures and Tables

**Table 1:** Demographics and clinical characteristics of study subjects (n = 41).

Variables	Value
Age (years, mean ± SD)	59.487 ± 10.406
Gender (N, %)	
Male	9, 23.08%
Female	30, 76.92%
Eyes (N, %)	
Right	18, 46.15%
Left	21, 53.85%
AL (mm, mean ± SD)	24.318 ± 1.914
BCVA (logMAR, mean ± SD)	
Preoperative	0.929 ± 0.494
2 months postoperative	0.415 ± 0.294
6 months postoperative	0.311 ± 0.283
Classification of IMH	
Small (N, %)	8 (20.51%)
Medium (N, %)	8 (20.51%)
Large (N, %)	23 (58.97%)

AL, axial length; BCVA, best correct visual acuity.

**Table 2:** Reduction of inner and outer retinal thickness in each quadrant in the patients with DONFL during the different postoperative periods.

	Temporal	Nasal	*p1*	Superior	Inferior	*p2*
**ORL**						
**2 months**	28 (−1, 54)	32 (−3, 56)	1.000	20 (0, 46)	23 (−3, 58)	1.000
**6 months**	34 (11, 67)	35 (0, 57)	1.000	25 (6, 50)	33 (2, 57)	1.000
**IRL**						
**2 months**	22 (14, 32)	7 (−6, 20)	< 0.001 †	11 (0, 21)	13 (−1, 25)	0.171
**6 months**	25 (18, 37)	10 (−1, 25)	< 0.001 †	17 (3, 27)	17 (5, 29)	0.077

DONFL, dissociated optic nerve fiber layer; ORL, outer retinal layer; IRL, inner retinal layer. The *p1* was obtained by Bonferroni correct test comparing the data of temporal and nasal in GEE analysis, and *p2* was obtained by Bonferroni correct test comparing the data of superior and inferior in GEE analysis.

**Table 3:** The correlation between BCVA and the reduction of retinal thickness in each quadrant in the patients with DONFL 6 months after surgery, using Spearman’s correlation test.

	Temporal	Nasal	Superior	Inferior	Mean
**Outer retina layer**					
***r*-value**	0.161	0.14	0.071	0.045	0.093
***p*-value**	0.328	0.394	0.666	0.786	0.575
**Inner retina layer**					
***r*-value**	-0.063	0.104	0.038	0.103	0.069
***p*-value**	0.704	0.529	0.816	0.534	0.678

## Data Availability

The datasets used and analyzed during the current study are available from the corresponding author upon reasonable request.

## Supplementary Materials

The following supporting information can be downloaded at: https://www.mdpi.com/article/10.3390/jpm13020255/s1, Table S1: The area of RNFL in the patients with DONFL during postoperative follow-up; Table S2: The retinal thickness of each layer in each quadrant in the patients with DONFL during postoperative follow-up; Table S3: The baseline of inner and outer retinal thickness in each quadrant in the patients with DONFL.
