# Supplementary material for: Analysis of Retinal Microstructure in Eyes with Dissociated Optic Nerve Fiber Layer (DONFL) Appearance following Idiopathic Macular Hole Surgery: An Optical Coherence Tomography Study
Source: J Pers Med. 2023 Jan 30;13(2):255. doi: 10.3390/jpm13020255 (PMC9963747; doi:10.3390/jpm13020255)
Supplement: Supplementary file 1 [file jpm-13-00255-s001.zip › jpm-2154698-supplementary.pdf]

Supplemental Table S1. The area of RNFL in the patients with DONFL during postoperative follow-up

Time period	Preoperative	2 months	6 months	*p1* value	*p2* value
**RNFL (pixel)**	2945.436±559.580	3048.222±572.067	2841.256±590.553	0.623	0.463

p1 was obtained by Bonferroni correct test comparing the data preoperatively and 2-month postoperatively.

p2 was obtained by Bonferroni correct test comparing the data preoperatively and 6-month postoperatively.

p1 and *p2* was based on repeated measures ANOVA

Supplemental Table S2. The retinal thickness of each layer in each quadrant in the patients with DONFL during postoperative follow-up

	Preoperative	2 months	6 months	*p*
**Mean (μm)**				
**TRL**	370.365±59.043	322.788±42.214	310.910±29.162	< 0.001†
**ORL**	256.635±49.588	224.083±32.690	217.333±18.565	< 0.001†
**IRL**	113.731±21.699	98.660±16.632	93.577±16.807	< 0.001†
**Temporal (μm)**				
**TRL**	361.026±61.052	299.179±43.380	286.333±30.369	< 0.001†
**ORL**	251.231±48.023	215.282±29.004	207.231±17.883	< 0.001†
**IRL**	109.795±27.680	83.897±17.615	79.103±16.158	< 0.001†
**Nasal (μm)**				
**TRL**	380.513±60.896	339.615±47.260	330.923±33.400	< 0.001†
**ORL**	268.128±56.700	234.026±37.470	229.000±20.480	< 0.001†
**IRL**	112.385±17.962	105.538±18.735	101.923±19.746	0.008†
**Superior (μm)**				
**TRL**	369.923±56.575	329.077±40.342	318.051±33.702	< 0.001†
**ORL**	255.026±50.281	225.436±36.542	219.692±28.385	< 0.001†
**IRL**	114.897±16.757	103.564±16.461	98.359±18.635	< 0.001†
**Inferior (μm)**				
**TRL**	370.000±62.706	323.282±40.240	308.333±24.894	< 0.001†
**ORL**	252.154±50.862	221.590±30.000	213.410±15.280	< 0.001†
**IRL**	117.846±29.829	101.641±19.005	94.923±18.056	< 0.001†

DONFL: Dissociated optic nerve fiber layer; TRL: total retinal layer; ORL: outer retinal layer, IRL: inner retinal layer.

p was based on repeated measures ANOVA

Supplemental Table S3. The baseline of inner and outer retinal thickness in each quadrant in the patients with DONFL.

	Temporal	Nasal	*p1*	Superior	Inferior	*p2*
**ORL**	251.231±48.023	268.128±56.700	< 0.001	255.026±50.281	252.154±50.862	1.000
**IRL**	109.795±27.680	112.385±17.962	1.000	114.897±16.757	117.846±29.829	1.000

DONFL: Dissociated optic nerve fiber layer; ORL: outer retinal layer, IRL: inner retinal layer. p1 was obtained by Bonferroni correct test comparing the data of temporal and nasal in GEE analysis. p2 was obtained by Bonferroni correct test comparing the data of superior and inferior in GEE analysis.
